# Building Development and Roads: Implications for the Distribution of Stone Curlews across the Brecks

**DOI:** 10.1371/journal.pone.0072984

**Published:** 2013-08-30

**Authors:** Ralph T. Clarke, Durwyn Liley, Joanna M. Sharp, Rhys E. Green

**Affiliations:** 1 School of Applied Sciences, Bournemouth University, Poole, Dorset, United Kingdom; 2 Footprint Ecology, Wareham, Dorset, United Kingdom; 3 Conservation Science Group, Department of Zoology, University of Cambridge, Cambridge, United Kingdom; 4 Royal Society for the Protection of Birds, Sandy, Bedfordshire, United Kingdom; University of California, Berkeley, United States of America

## Abstract

**Background:**

Substantial new housing and infrastructure development planned within England has the potential to conflict with the nature conservation interests of protected sites. The Breckland area of eastern England (the Brecks) is designated as a Special Protection Area for a number of bird species, including the stone curlew (for which it holds more than 60% of the UK total population). We explore the effect of buildings and roads on the spatial distribution of stone curlew nests across the Brecks in order to inform strategic development plans to avoid adverse effects on such European protected sites.

**Methodology:**

Using data across all years (and subsets of years) over the period 1988-2006 but restricted to habitat areas of arable land with suitable soils, we assessed nest density in relation to the distances to nearest settlements and to major roads. Measures of the local density of nearby buildings, roads and traffic levels were assessed using normal kernel distance-weighting functions. Quasi-Poisson generalised linear mixed models allowing for spatial auto-correlation were fitted.

**Results:**

Significantly lower densities of stone curlew nests were found at distances up to 1500m from settlements, and distances up to 1000m or more from major (trunk) roads. The best fitting models involved optimally distance-weighted variables for the extent of nearby buildings and the trunk road traffic levels.

**Significance:**

The results and predictions from this study of past data suggests there is cause for concern that future housing development and associated road infrastructure within the Breckland area could have negative impacts on the nesting stone curlew population. Given the strict legal protection afforded to the SPA the planning and conservation bodies have subsequently agreed precautionary restrictions on building development within the distances identified and used the modelling predictions to agree mitigation measures for proposed trunk road developments.

## Introduction

The UK human population is projected to increase from 62.3 million in 2010 to 70 million to 2027 [1]. Government policy is promoting new development as a means of contributing towards economic growth. This growth requires local planning authorities to develop and implement plans for the spatial allocation of additional housing and associated infrastructure.

Within the UK, sites designated as Special Areas for Conservation (SAC) or Special Protection Areas (SPA) are part of the Natura 2000 network of protected sites, designated for their nature conservation interest. Such sites are subject to strict legal protection, enshrined within UK law through the Habitat Regulations. Plans or projects likely to have a significant effect on such sites must be subject to an Appropriate Assessment, requiring the competent authority to ascertain that the implementation of the plan / project will have “no adverse effect on the integrity of European-protected sites” (unless there are imperative reasons of overriding public interest). In order to be confident of no adverse effect, scientific evidence is necessary to help inform, minimise and resolve potential conflicts between the needs for development and nature conservation requirements.

The Breckland Special Protection Area (SPA) in eastern England qualifies under Article 4.1 of the European Birds Directive (79/409/EEC) in part because it supports a large population of stone curlews *Burhinus* *oedicnemus* . Stone curlews are summer visitors which breed in southern England and winter in France, Spain, Portugal and North and West Africa [2]. Their breeding sites are restricted to areas with sandy and stony soils and most breeding pairs are found in the Breckland region of East Anglia and Salisbury Plain where they nest on sparsely vegetated ground on short semi-natural grassland, heaths and spring-sown arable fields [3].

In 1998 (the year given in the SPA citation), the SPA supported some 142 pairs of stone curlews, some 75% of the UK population; whilst by 2008, the Brecks area held 216 breeding pairs comprising just over 60% of the current UK total. The SPA covers over 39,000 ha and includes farmland as well as semi-natural habitats. It covers parts of several planning districts (Breckland, Forest Heath, St. Edmundsbury Borough, King’s Lynn and West Norfolk Borough).

There is evidence that housing can have negative effects on the nature conservation interest of nearby habitats, especially heathland [4–6]. Studies of disturbance by humans and vehicles of nesting stone curlews [7] found that incubating birds respond to potential disturbance events from approaching vehicles, walkers and especially approaching dog walkers at distances of about 400-500m. Other work has shown a clear avoidance by nesting stone curlews of otherwise suitable habitat adjacent to major roads [3]. Road traffic data has been used to further explore this avoidance and analysis [8] has shown an effect of major roads, with the avoidance increasing over time, in parallel with traffic flows.

The stimulus for this research was the need to assess the relationship between housing, major roads and stone curlew nest spatial distributions in order to predict the effect of future housing developments and associated road traffic increases within the Brecks on nest distribution [9]. Regional development plans in 2008 included a minimum of 15,200 new houses to be built in Breckland District by 2021 [9]. The work formed part of the evidence to inform the affected district councils’ strategic planning policies. The UK Royal Society for the Protection of Birds (RSPB) has collected data on stone curlew nest locations across the entire area annually since 1988, with others contributing data since 2000. This provided a unique opportunity to explore stone curlew breeding distribution in relation to housing and roads. We used the nest distribution data in conjunction with data on the spatial distribution of housing, roads and road traffic flows to assess whether housing and roads appear to influence the distribution of stone curlew nests.

## Methods

### Bird Data

Data on the specific location of stone curlew nests (to the nearest 50m) within the Breckland region 1988-2006 were provided by the RSPB and others. Nests were located by visual scanning of areas used for nesting in previous years and other potential habitat, and watching the parents from a distant vantage point. Systematic searches for other pairs were also carried out in April and May by playing taped calls at night and returning by day to check areas from which birds were heard to call in response, and also in response to reports from land owners and managers [10]. The proportions of nests (and breeding pairs) detected in each year have not been estimated. However, Day [8], using a method based upon re-sightings of colour-marked adults [10], found that the surveys detected over 90% of adults of breeding age. The proportion of breeding birds detected might be somewhat higher than this because not all birds old enough to breed in a given year actually do so [11]. The occurrence of foot and mouth disease in 2001, with associated restrictions on access for survey, resulted in an incomplete dataset for that year, which was therefore removed from analyses. All observed nests between 1988 and 2006 were mapped in a GIS (MapInfo 9.0 was used throughout).

### Study Area and Suitable Habitats

**Figure 1:**
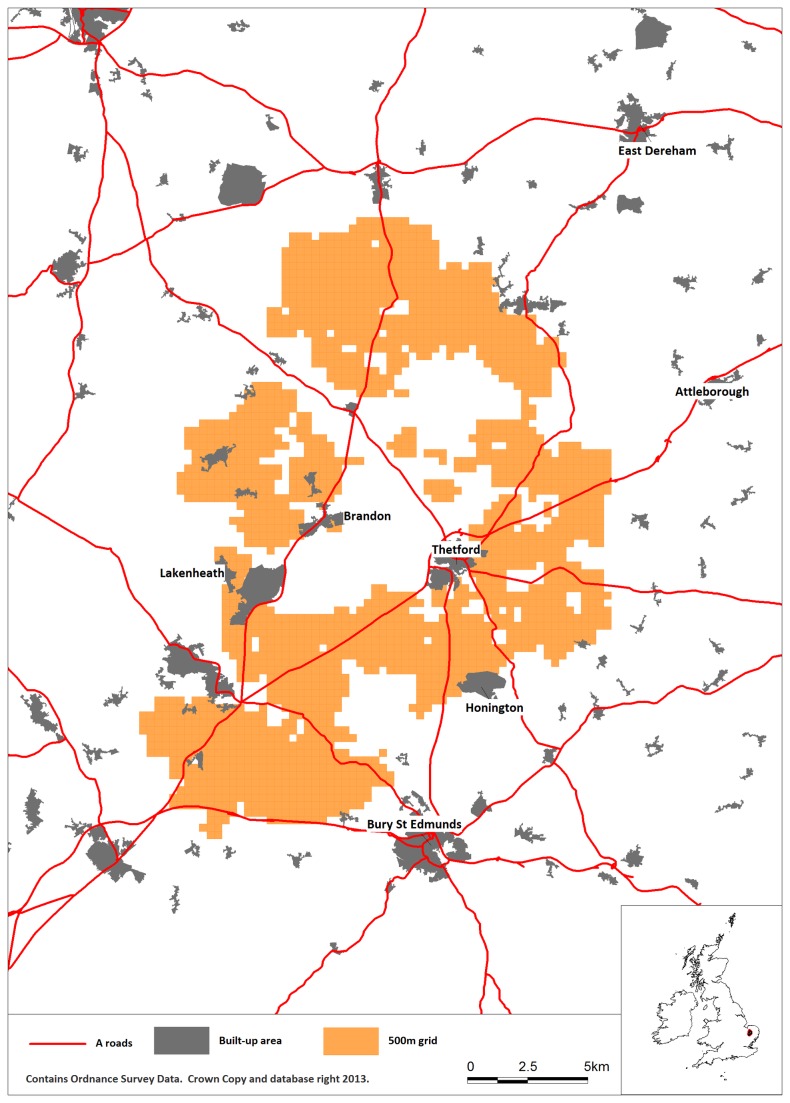
Map of overall study area (red line) showing the main settlements, main roads and the area (of 500m cells) containing potentially suitable habitat surveyed for stone curlew nests.

The broad study area was defined to include a 5 km buffer drawn around the convex polygon encompassing all nest points (Figure 1). Within this study area suitable stone curlew habitat was identified using results from a previous analysis [3]. Their study showed that the boundaries of the geographical range of breeding stone curlews in southern England and the location of nest sites within the range are both associated with certain soil associations. We used soil data provided under license by the National Soil Resources Institute (NSRI) to restrict our analyses to parts of Breckland with these associated soil types, namely rendzinas (NSRI soil code 3.4), brown calcareous sands (5.2) and brown sands (5.5).

Stone curlews nest at the highest densities on short semi-natural grassland, but they occur at lower density on spring-sown arable fields [3]. Semi-natural grassland occurs in the predominantly arable farmland and commercial forestry landscape of Breckland as nature reserves, Sites of Special Scientific Interest and military training areas. The suitability of semi-natural grasslands for stone curlews varies very substantially among sites and within a site among years. This variation is strongly associated with differences in vegetation height, which are largely caused by differences and changes in grazing pressure, especially by rabbits *Oryctolagus cuniculus* [3,12]. Stone curlew breeding sites on semi-natural land are associated with short, sparse vegetation and tend to be abandoned when the vegetation height exceeds about 2 cm [11,13]. For this reason, crude classifications of vegetation type are not a good predictor of the suitability of semi-natural habitats for stone curlews. Annual ground-based measurements of vegetation structure or appropriately adjusted measurements of vegetation cover using satellite imagery are needed for this [11,13]. Detailed assessments of this kind have not been made. Breeding stone curlews on semi-natural sites are also affected by disturbance from public access and military training [7], the levels of which have also not been measured systematically for Breckland semi-natural grasslands. Arable fields vary in their suitability for stone curlews according to their crop and soil type, and to some extent in relation to their proximity to foraging habitats [3]. Disturbance by public access and other factors is lower and much less variable among arable sites than is the case for semi-natural grassland. We reduced the effect of soil type by restricting our analysis to areas with suitable soil types. Suitable spring-sown crop types for stone curlews are relatively common in Breckland (40-50% of the arable area [3]), and the arable crop grown in a given field changes regularly because of crop rotation. Therefore, we consider that, when averaged over the long time periods considered in our analysis, spatial variation in habitat suitability due to factors other than proximity to buildings and roads is less for arable farmland than for semi-natural grassland. Most of our analysis was therefore focussed on arable farmland. GIS data on land use were provided by the RSPB, from a single ground-based survey in 1997, when all arable fields, grassland fields and SSSIs were surveyed and mapped onto 1:25000 Ordnance Survey maps. As the main patterns of land use within the Breckland region are unlikely to have changed much throughout the period 1988 to 2006, these data were used to split the area within the study area on suitable soils in all years into those on arable land (total area 284.6 km^2^) and those on the much smaller area of semi-natural grassland/SSSIs (119.7 km^2^).

### Buildings Data

The location of all buildings within the study area at the end of the study period was extracted from Ordnance Survey MasterMap data (dated 2007). It was not possible to distinguish between residential and commercial buildings. In order to identify which of these buildings had been built since 1988, successful planning applications over the study period, acquired from the district councils within the study area, were located within the filtered MasterMap data. Only planning applications for at least three new properties were located for back-calculation. For Breckland District the information was acquired directly from the planning department, while for all other district councils their online planning database was used to extract the information. We assumed a standard 12-month time lag from time of planning application to building commencing. We were therefore able to successively remove each year’s new buildings from the most recent 2007 MasterMap data and back-calculate maps of building areal distribution for each year back to 1988 within the GIS.

Within the GIS, a subset layer which we refer to as “settlements” was derived from the most recent building distribution, by including all towns and villages but excluding farm buildings, small developments (generally < 5 buildings) and isolated or lone buildings. Towns and villages that were used include, but are not limited to, Thetford, Brandon, Lakenheath, Weeting, Feltwell, Mundford, Watton, Swaffham, Hockham, Rushford, Hengrave and Mildenhall.

### Road and Traffic Data

Spatial data for all main roads (both trunk (generally fast long-distance) roads and non-trunk A-class roads) within the study region were also extracted from OS MasterMap (Figure 1). Traffic data was acquired from the TRADS system (http://trads.hatris.co.uk/), part of the Highways Agency (HA). Bi-directional continuous hourly traffic flow data for 2002-06, available for each of a number of sections of the A11 and A14 trunk roads crossing the study region, was translated into month-by-month average daylight, darkness and total daily traffic flows. Because stone curlew tend to nest between March and August [3], only average daily traffic flow data over this period of months was used in subsequent stone curlew modelling.

### Statistical Analyses

#### Nest Density - Distance to Nearest Settlement

Initial analyses assessed the variation in nest density with distances to the nearest ‘settlement’. All points within the study area were classified into 500m bands of distance from the nearest ‘settlement’ building (up to a maximum of 4000m) and then the total area of suitable habitat, stone curlew nest number and thus average nest density within each distance band were calculated for each year. We chose 500m bands to have sufficient nests in a band to have adequate statistical power to detect reduced nest densities relative to land further away from the potential disturbances. This does not assume a linear response across the whole distance range assessed and our later models allow for non-linear monotonic responses. Arable and semi-natural grassland/SSSI habitats on suitable soils were analysed separately.

Sequential chi-square goodness of fit (GOF) tests were used to test whether nest density within a particular 500-m distance band was statistically significantly less than the average nest density in the combined area of suitable land at all greater distances (up to 4000m). Each Chi-square GOF test compared the observed number of nests in the two classes of distance band with the numbers expected based on their relative areas and their observed combined number of nests. First, nest density in areas with 500m of settlements was compared with average nest density in all combined more distance areas; then areas and nests within 500m of settlements were excluded and nest densities within 500-1000m of the nearest settlement were compared with average density in all combined more distant areas on suitable arable soils, and so on. The highest distance band at which there were still statistically significant differences (i.e. Chi-square GOF test p < 0.05) in average nest density between this and higher distance bands was used to indicate the maximum distance at which we can detect a strong association of nest density with proximity to settlements. This analysis was done first using the combined year nest numbers in each distance band. It was then repeated for each year separately to assess whether any effect has persisted through time and is detectable within individual breeding years; acknowledging the expected lower statistical power to detect effects when there are relatively low total nests per year.

#### Nest Density - Distance to Nearest Road

A similar type of exploratory analysis was used to assess nest density in relation to distance from A-roads. Using the MasterMap spatial road network data, for each individual A-road (trunk and non-trunk) within the study region, buffers around the road were drawn at regular 500m intervals from the road (up to a limit of 3000m) and within each buffer we calculated the area of suitable arable land and the number of stone curlew nests on that land. Successive Chi-square tests were repeated to test for differences in nest density with distance from the nearest trunk road as described above for the analysis of the effect of proximity to nearest settlement.

#### Statistical Modelling in relation to nearby Buildings, Roads and Traffic

The simple analyses described above are not entirely satisfactory because they consider the effects of proximity to buildings and roads separately and ignore possible confounding effects between the two variables. We therefore performed an analysis to take both variables into account together. This was only done for arable land, because of the spatial variation in habitat quality on semi-natural grassland, as discussed above. We divided the study area into 500 x 500 m square cells based upon the Ordnance Survey grid (Figure 1). This grid cell size was chosen to make the subsequent nest distribution spatial modelling computationally tractable, while still giving adequate accuracy in terms of distances from nests to buildings and roads. Amongst the 2142 cells with some area (*A*_*i*_) of arable habitat on suitable soil types (average 13.3 ha. per 25 ha. cell), 24.2% held a nest in one or more years. Only 3.8% (1463) of all cell-year combinations had any nests. Of these, the vast majority (84%) had only one nest, while there were 197, 34 and 7 occasions with 2, 3 or 4 or more nests respectively.

We measured distance from each 500m cell to (i) the nearest settlement (as defined above), (ii) the nearest A-road and (iii) the nearest Trunk A-road. Shortest distances were set to zero if the feature was present within the 500m cell.

The data on annual nests and distances to settlements and roads for each 500m cell, together with the analysed overall annual nests and habitat areas in each 500m distance band from nearest settlement or trunk road are available from: http://www.footprint-ecology.co.uk/publications_and_downloads.html

#### Distance-weighted kernel variables for local buildings and road traffic

A grid of 50 x 50 m square cells was also constructed and for each 50-m cell we extracted the area of buildings in each year. The following road/traffic variables were measured for each 50m grid cell, (i) the presence (scored 1) or absence (0) of a trunk A-road within the cell, (ii) the presence (1) or absence (0) of a non-trunk A-road and (iii) the volume of traffic along any section of trunk A-road passing through the cell. The traffic volume variables entailed the March–August monthly average daylight, darkness and total daily traffic flows averaged across the period 2002-06.

For each 500-m cell (*i*), we calculated the distance *D*_*ik*_ to each 50m cell *k*. Each 500m and 50m cell is represented as a polygon and the distance *D*_*ik*_ is the shortest distance between the two polygons, so all 50m cells either inside or touching the 500m cell are given a distance *D*_*ik*_ of zero.

For the kth 50-m cell, let *V*_*k*_ denote the value of the buildings/road/traffic predictor variable for that cell. Although it is not known how any effect of buildings or roads on stone curlews diminishes with distance, we used a half-normal kernel weighting determined by a standard deviation (SD) *s*, where *s* ranged from 250m to 2000m, in steps of 250m. The weight *W*_*ik*_ given to a 50-m cell *k* at a distance *D*_*ik*_ from 500m cell *i* was *W*_*ik*_ = *exp*(-(*D*_*ik*_/s)^2^). Then the value *X*_*Vi*_ of predictor variable *X*_*V*_ for 500m cell *i* is a weighted sum of the *V*_*k*_ values across all cells, namely *X*_*Vi*_ = ∑_k_ *W*_*ik*_ *V*_*k*_. When *D*_*ik*_ = 0, the weight is 1.0, at distances *D*_*ik*_ of *s* and 2s, the weighting *W*_*ik*_ is reduced to 0.368 and 0.018 respectively.

For computational efficiency and tractability, the summation is limited to 50-m cells within two standard deviations (s) of the 500-m cell *i* (i.e. where *D*_*ik*_ ≤ 2s). Larger values of *s* cause the predictor variable *X*_*i*_ to be influenced by the amount of buildings, roads and traffic over greater distances. We called the variables obtained by the kernel weighting procedure “local densities”, where the adjective “local” refers to the region defined by *s* within which the amount of buildings, roads or traffic influences stone curlew nest density in a focal cell.

#### Optimising model selection

Generalised linear modelling (GLM) analyses [14] were used to relate each of these half-normal kernel weighted buildings (*X*_*H*_) and road/traffic (*X*_*R*_) local density variables to the stone curlew nest density in each 500-m cell with the aim of finding the distance weightings *s* at which relationships were strongest. Modelling nest density per unit area of suitable land rather than merely presence/absence per 500m cell enabled any derived models to be used to predict the effects of proposed increases in housing (and/or road traffic) on stone curlew nest density on the suitable land and thus nest numbers. Specifically, we fitted quasi Poisson log-linear GLM models with two general forms:

(i) non-temporal model for the total number (*N_i_*) of stone curlew nests in 500m cell *i* in either a single year or 4-5 year period:

$$\log_{e}N_{i}=\log_{e}A_{i}+\alpha +\beta_{Hi}X_{Hi}+\beta_{R}X_{Ri}$$ (1)

where *A*_*i*_ = Area (in hectares) of arable land on suitable soil types in the 500m cell *i* (model offset term), *X*_*Hi*_ and *X*_*Ri*_ = variables representing (period-averaged) nearby buildings and road/traffic local densities for cell *i* (using independently selected values of *s* for buildings and roads) and α, *β*_*H*_ and *β*_*R*_ are the model parameters to be estimated

(ii) whole 1988-2006 dataset model for the number (*N_iy_*) of stone curlew nests in 500m cell *i* in year *y*:

$$\log_{e}N_{iy}=\log_{e}A_{i}+\alpha_{y}+\beta_{Hi}X_{Hiy}+\beta_{R}X_{Riy}$$ (2)

where *X*_*Hiu*_ and *X*_*Riy*_ = value of the buildings and road/traffic local density variables for cell *i* in year *y* and *α*_*y*_ = factor representing a year-specific intercept.

Initial model selection was based on fitting model equation 2 with Poisson errors using one buildings variable (*X*_*H*_) and one road/traffic variable (*X*_*R*_) measuring nearby local density of either A-roads, trunk roads or average daily traffic levels on the trunk roads. Models were fitted using all possible combinations of *s* (250-2000m) for the buildings and road/traffic variables. Additional candidate variables included distance to nearest settlement, distance to nearest Trunk road and distance to nearest A-road (including trunk roads). The relative fits of these alternative two-variable (one buildings, one road/traffic) GLM models were assessed and compared by their values for Akaike Information Criterion (AIC = -2(Log Likelihood + number of fitted parameters); smaller is better). Effects of any extra-Poisson residual dispersion in nest numbers were allowed for by re-fitting models using quasi-Poisson errors which increases the Poisson-likelihood-based standard errors (SE) of the regression model coefficients {*β*_*H*_, *β*_*R*_} by a factor (√*q*), where *q* is the estimate of the Poisson variance dispersion parameter [14]. However, Poisson maximum likelihood and quasi-Poisson maximum likelihood give the same model fit and parameter estimates, so it was valid to use AIC values to compare these model fits. GLM models were fitted using the glm function in the R software package (version 2.15.2).

#### Allowing for spatial autocorrelation

Spatial autocorrelation of residuals can influence the reliability of any such statistical models relating environmental factors to species’ distributions, both in terms of accuracy of statistical significance of effects and accuracy of the effect sizes (i.e. model coefficients). Dormann et al. [15] discussed a wide range of existing methods to try to allow for spatial autocorrelation; based on their simulated data (with known spatial correlation of errors) they concluded that effects of environmental factors on species occurrences are consistently under-estimated by auto-covariate methods (whereby the value of dependent variable y at a point is assumed to be influenced by a weighted-average of the y values of geographically-close observations). As we intended to use our fitted models for prediction to new buildings and increased traffic effects, we avoided using such auto-covariate models.

We used Generalised Linear Mixed Models (GLMM), which are an extension of Generalised Least Squares (GLS) to cope with errors/residuals which are both non-normal (such as our (quasi) Poisson nest count errors) and non-independent (e.g. spatially correlated, as here). Bolker et al. [16] provide a useful discussion of the range of different software options to fit GLMM in general, but conclude that no single approach is optimal for all problems but depends on the importance of hypothesis testing, accurate unbiased parameter estimating and prediction. Beale et al. [17] used a wide range of simulated data with varying strengths and varying spatial scales of exponential-decay spatial auto-correlation to assess the accuracy (bias and sampling precision) of various models and fitting methods on parameter estimates and hypothesis test Type I error rates. They concluded that as spatial autocorrelation increased, ignoring it by fitting Ordinary Least Squares (OLS) models led to over-estimation of (absolute values of) predictor variable parameter estimates and much too high Type I error rates. In contrast, GLS models, even fitted with a slightly different (spherical) form of autocorrelation structure was one of several model methods providing “generally good overall performance” [17]. Unfortunately their study was based solely on normally distributed correlated errors, well fitted by GLS; however GLMM are the extension to GLS for non-normal errors.

We fitted GLMM extensions of the non-temporal GLM model (equation 1) involving buildings and road/traffic variables that included and allowed for a spatial auto-correlation (r) between model residuals which declined with distance *d* apart of nest observation cells in accordance with either an exponential decay (*r* = exp(-d/*w*) or Gaussian (*r* = exp((-d/*w*)^2^) function. Models parameters (including *w*) were fitted by maximising the penalised quasi-likelihood using the glmmPQL function of package MASS in R, which can incorporate a range of such spatial correlation structures. However, such model fitting using glmmPQL on our stone curlew nest data with 2142 nest cell observations (and their residual covariance structure) was slow (1-2 hours). Therefore GLMM were only fitted to the combinations of buildings and road/traffic variables which gave the best fits (minimum AIC) from the initial GLM analyses.

## Results

### Overview of Stone Curlew Population Trends

**Figure 2:**
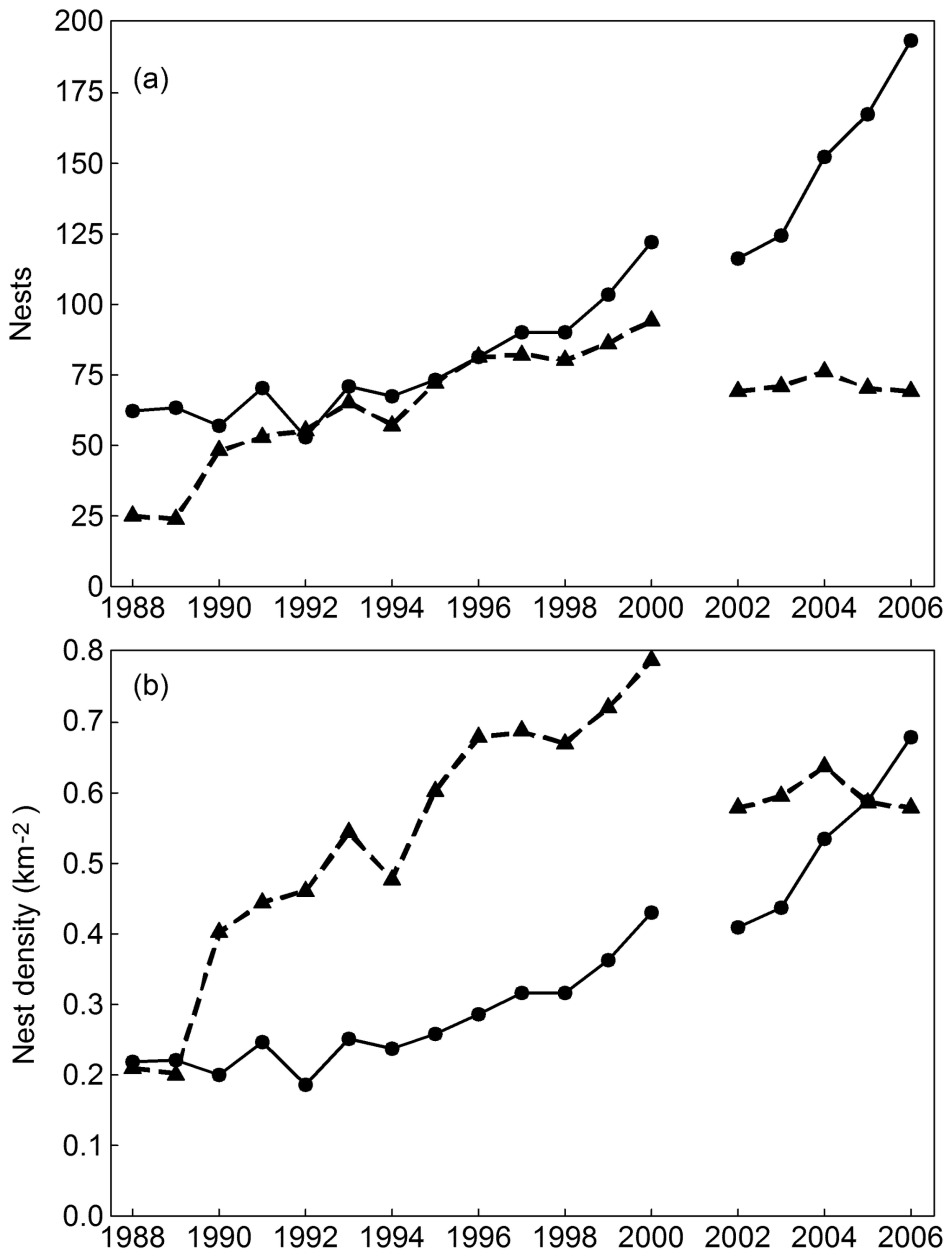
Stone curlew population trends 1988-2006. (a) nests per year and (b) nest density (km^-2^) on suitable arable land (solid) and semi-natural grassland/SSSI (dashed).

From 1988 to 2006 the total number of stone curlew nests found within the region has steadily increased from 87 in 1988 (62 on arable land; 25 on SSSI/semi-natural grassland) to 262 in 2006 (193 on arable land; 69 on SSSI/semi-natural grassland) (Figure 2(a)). However, since 2000, the number of nests found on semi-natural grassland/SSSI has been lower while on arable land the number has continued to increase. The number of nests on arable land is consistently greater than that on non-arable land, however this is largely due to the larger area of arable land (284.6 km^2^) available compared to that of semi-natural grassland/SSSI (119.6 km^2^). Nest densities at the start of the study period were about the same on both habitats but then during the early and mid-1990s, densities on the semi-natural grassland/SSSI increased faster reaching a peak in 2000. Average nest densities on arable land have steadily increased throughout the study period and by 2006 exceeded average densities in semi-natural grassland/SSSI (Figure 2(b)). It should be borne in mind here that much of the area classified as semi-natural grassland/SSSI in our analysis has ground layer vegetation which is too tall and dense to be suitable for stone curlews [11]. Stone curlew densities on the portion of the semi-natural grassland with a short sward would be much higher, but we cannot quantify the area of this subset on an annual basis.

To assess the strength of temporal correlation in distribution between successive years, we calculated the proportion of the 500m gird cells occupied by one or more stone curlew nests in one year that were also occupied the next year (i.e. re-occupied). Obviously any error in recording nest location to the nearest 50m will also contribute to some apparent change in nest location, but this is expected to be minor given the cell size. The median value of this measure of temporal auto-correlation was only 0.45, but it has increased over time from only 0.12 in 1988-89 to 0.92 in 2005-06 ). The observed increase in stone curlew local population totals (at least in terms of observed nests) may have reduced the choice of remaining unoccupied suitable territories, thus increasing the observed tendency for more cells to be occupied in consecutive years. However, especially in earlier years, there is considerable turnover and change in the precise areas which are used for nests each year, which indicates that the individual years’ data do provide useful extra information to assess any apparent observed relationship between stone curlew nests and distance to nearest settlement or roads and/or their traffic.

Nest Density - Distance to Nearest Settlement

**Figure 3:**
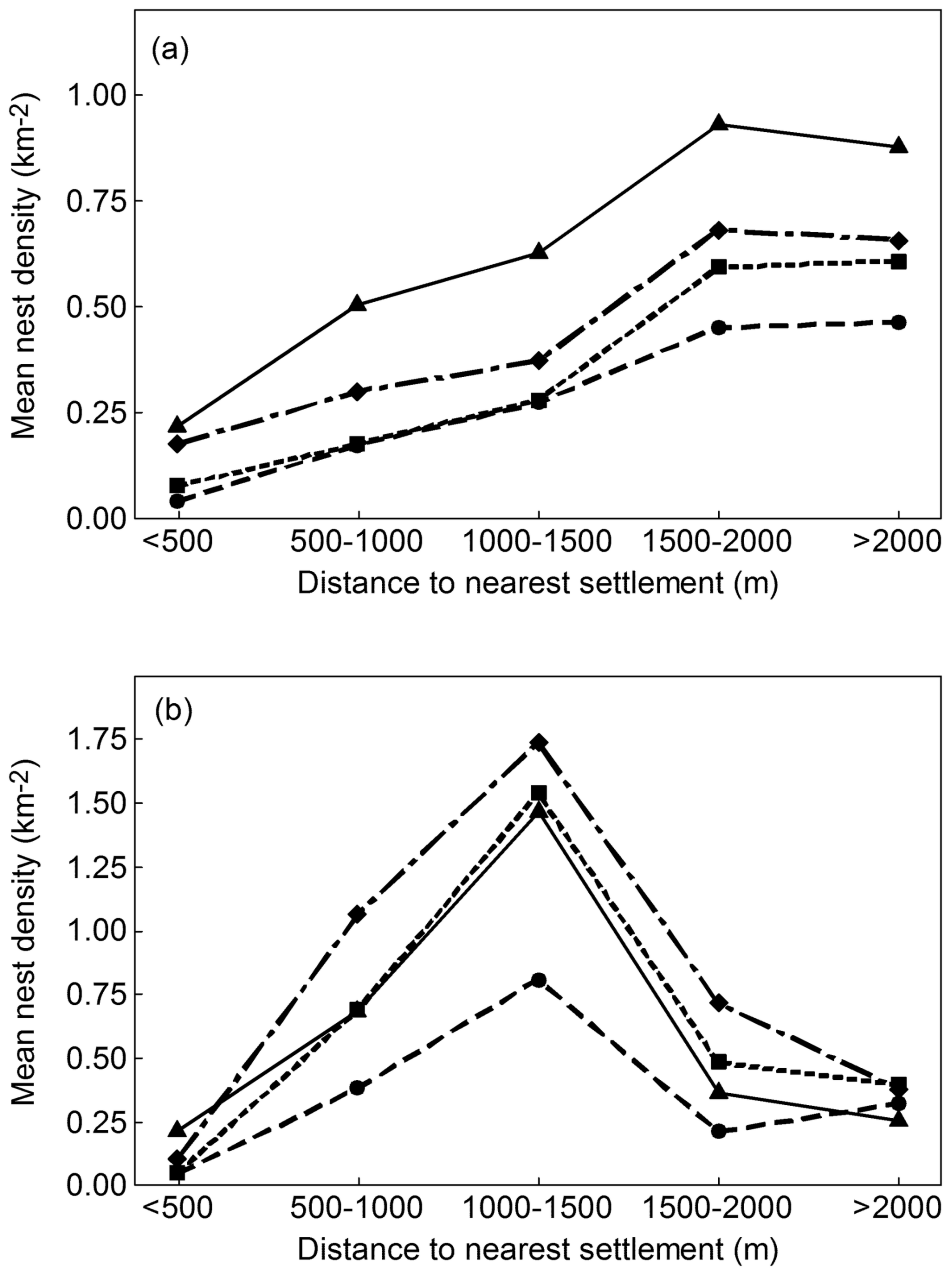
Nest density in relation to distance from settlements. Average density of stone curlew nests on (a) arable land and (b) semi-natural grassland and SSSI on suitable soils at different distance bands from the nearest settlement in each of the periods 1988-92 (circles), 1993-96 (squares), 1997-2000 (diamonds) and 2002-06 (triangles).

The average density of stone curlew nests per year on arable land of suitable soil type increased with distance from nearest settlement up to a distance of 1500m (Table 1). This pattern was present in every 4-5 year period of the study, even though stone curlew numbers on suitable arable land have tripled since 1988 (Figure 3(a)). In every year from 1988 to 2006, the stone curlew nest density (per ha of suitable arable land) was lower on land within 500m of the nearest settlement than on land either 500-1000m, 1000-1500m, 1500-2000m or 2000-4000m from the nearest settlement. Moreover, the nest density on suitable arable land within 500m of a settlement was statistically significantly (all *P*≤0.003) less than average density on all more distant suitable arable land in every year (Table 1).

**Table 1:** Annual density (km^-2^) of stone curlew nests on areas (km^2^) of suitable arable land within each band of distance (m) to the nearest "settlement”; together with the upper limit of the maximum distance band for which nest density is statistically lower (Chi-square test *P* value <0.05) than average nest density in the combined higher distance bands (*P* value for each test given in brackets).

		Distance band to nearest “settlement" (m)					
Period	Total nests (N)	<500	500-1000	1000-1500	1500-2000	2000-4000	Max distance (m) with lower nest density
All Years	1754	0.128 (<0.0001)	0.295 (<0.0001)	0.394 (<0.0001)	0.666 (0.797)	0.654	1500
1988	62	0.036 (<0.001)	0.211 (0.070)	0.233 (0.028)	0.415 (0.456)	0.549	500
1989	63	0.036 (<0.001)	0.223 (0.102)	0.217 (0.012)	0.533 (0.579)	0.431	500
1990	57	0.036 (<0.001)	0.149 (0.007)	0.350 (0.969)	0.385 (0.658)	0.314	1000
1991	70	0.059 (<0.001)	0.173 (0.002)	0.283 (0.015)	0.533 (0.635)	0.627	1500
1992	53	0.047 (<0.001)	0.111 (0.002)	0.283 (0.324)	0.385 (0.966)	0.392	1000
1993	71	0.071 (<0.001)	0.124 (<0.001)	0.317 (0.019)	0.651 (0.616)	0.549	1500
1994	67	0.059 (<0.001)	0.136 (<0.001)	0.267 (0.007)	0.474 (0.179)	0.745	1500
1995	73	0.083 (<0.001)	0.285 (0.361)	0.200 (0.003)	0.533 (0.902)	0.510	500
1996	81	0.095 (<0.001)	0.161 (<0.001)	0.333 (0.009)	0.711 (0.698)	0.627	1500
1997	90	0.142 (<0.001)	0.223 (0.002)	0.333 (0.009)	0.800 (0.179)	0.510	1500
1998	90	0.177 (0.007)	0.260 (0.029)	0.200 (<0.001)	0.770 (0.518)	0.627	1500
1999	103	0.201 (0.003)	0.322 (0.055)	0.333 (0.009)	0.651 (0.802)	0.706	500
2000	122	0.189 (<0.001)	0.396 (0.033)	0.617 (0.959)	0.503 (0.176)	0.784	1000
2002	116	0.118 (<0.001)	0.433 (0.123)	0.533 (0.379)	0.681 (0.800)	0.627	500
2003	124	0.201 (<0.001)	0.533 (0.970)	0.417 (0.072)	0.563 (0.298)	0.784	500
2004	152	0.201 (<0.001)	0.471 (0.004)	0.833 (0.806)	0.888 (0.342)	0.666	1000
2005	167	0.272 (<0.001)	0.483 (<0.001)	0.683 (0.021)	1.303 (0.057)	0.784	1500
2006	193	0.284 (<0.001)	0.607 (0.003)	0.667 (<0.001)	1.214 (0.302)	1.529	1500
Area (km^2^) (%)		84.6 (30%)	80.7 (28%)	60.0 (21%)	33.8 (12%)	25.5 (9%)	

The furthest 500-m distance band at which there were still statistically detectable (i.e. Chi-square test *P* < 0.05) differences in average nest density between this and the combined higher distance bands suggests the maximum distance at which we can detect an effect (or association) of buildings with nest density, when the data are subdivided by year. In eight of the 18 years, statistically significant lower nest densities were detectable for areas within 1000-1500m of the nearest settlement compared to areas further from any settlement (Table 1). In four other years the maximum distance with detectable reduction in nest density was 500-1000m and in the remaining six years, statistically significant differences were only detectable for the area up to 500m from the nearest settlement. The maximum distance from nearest settlement with detectably lower stone curlew nest densities did not change systematically with time (Table 1), even though total stone curlew numbers on the arable land have tripled over the past two decades. Average density in the 1500-2000 m band was not significantly different from that at greater distances, overall or within individual years; this appears to be more due to lack of effect beyond 1500m than lack of statistical power due to insufficient arable land and nest numbers (Table 1, Figure 3(a)).

**Figure 4:**
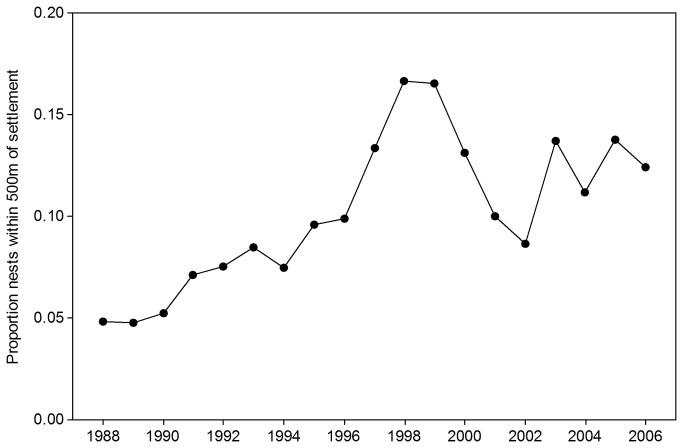
Proportion of nests on suitable arable land which occur within 500m of the nearest settlement.

There is some evidence that as the population size of stone curlews has increased over time relatively more birds have been nesting near buildings. The proportion of all nests on suitable arable land in the study area which is within 500m of the nearest settlement has steadily increased from around 5% in 1988-90 to peak at 16-17% in 1998-99 and reduced slightly to 11-14% since 2003 (Figure 4). However these percentages are still much less than the 30% expected from the proportion of all suitable arable land in the study region which is within 500m of the nearest settlement. Increases in total nest numbers over time provide increasing statistical power to detect the same proportional reduction in nest density close to settlements; thus the strength of avoidance of areas very close to settlements may have decreased slightly, but it remains and is still detectable statistically (Table 1).

There was much less semi-natural grassland/SSSI within the study region and it occurred in fewer patches; this led to much smaller areas in each 500m distance band from settlements (31.3, 20.0, 18.8, 17.1 and 15.6 km^2^ in the five bands out to 2500m). Therefore nest numbers in each distance band were combined into 4-5 year periods to provide adequate numbers for the sequential Chi-square tests. In each period, average nest density was lowest on land within 500m of a settlement, higher in the 500-1000m band and higher still in the 1000-1500m band, but thereafter declining for the available areas more than 1500m from any settlement (Figure 3(b)). This unusual pattern is likely to be due to variation among the few large blocks in the quality of semi-natural habitat, and especially the relatively poor quality of much of the habitat in the largest semi-natural fragment which is a large military training area and contains a large area distant from settlements. This highlights the unavoidable problem that the spatial distribution of stone curlew suitable soils and habitat (assumed constant of necessity over the study period) occurs in patches of varying size and quality which introduces an unknown element of spatial auto-correlation in the data. This may influence the true statistical significance of such Chi-square tests, but not the observed pattern of relationships of average nest density with distance from nearest settlement.

Nest Density - Distance to Trunk and A-Roads

**Figure 5:**
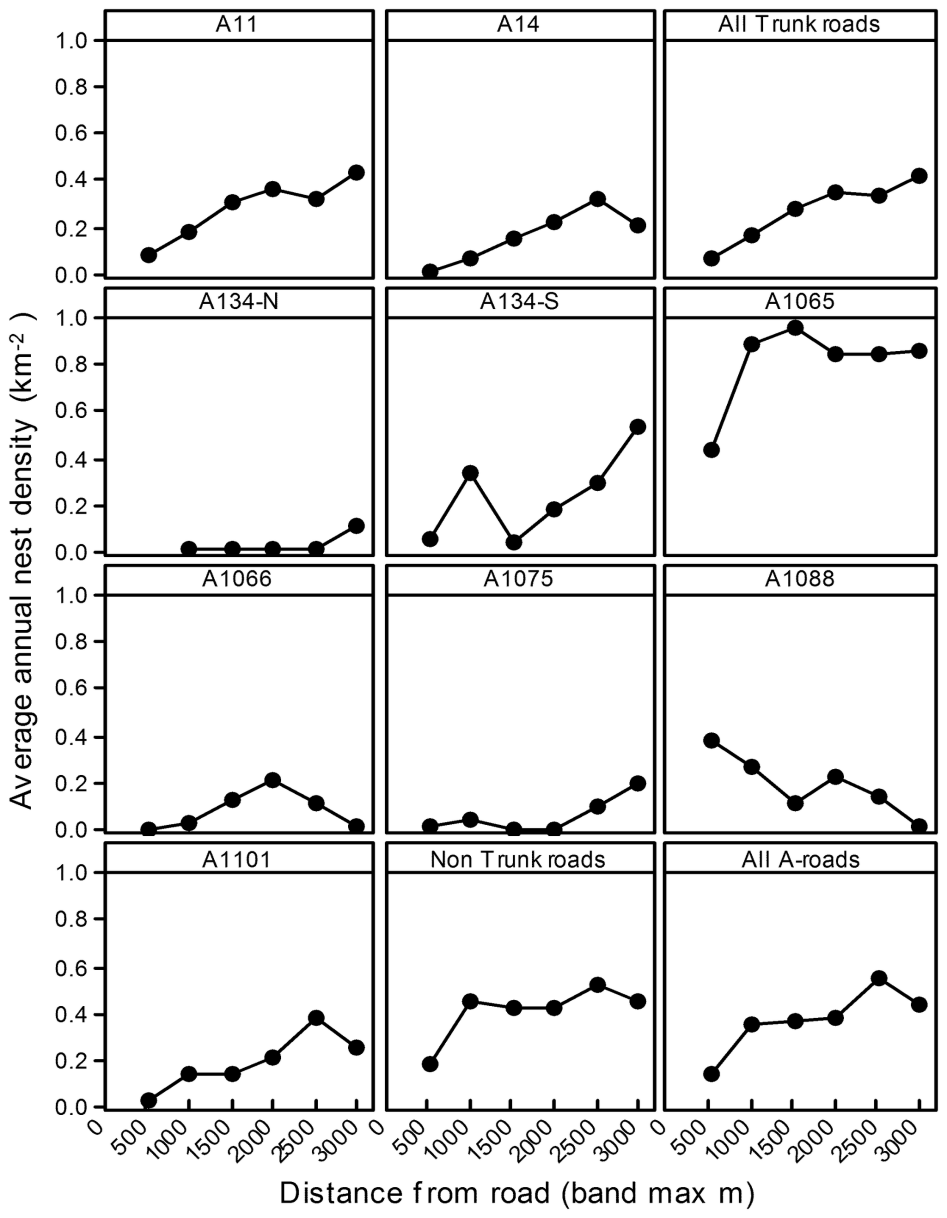
Nest density in relation to distance from A-roads. Average (1988-2006) annual density (km^-2^) of stone curlew nests on arable land at different distance bands from individual A-roads, from any Trunk road (A11,A14), from any non-Trunk road and from any A-road.

The average nest density on suitable arable land over the study period 1988-2006 appears to increase with distance from each trunk A-road (A11, A14) and from the nearest trunk road, up to distances of at least 1500m (Figure 5 top row). However, on the other (non-trunk) A-roads, the pattern is inconsistent. Densities appear lower on suitable arable land within 500m of the A1065 and A1101 roads, but decline with distance from the A1088 over the 10km stretch that is within the study region (Figure 5). Overall, when suitable arable land is classified into distance bands from the nearest non-trunk road or from the nearest A-road (including trunk roads), average nest density is lower when within 500m, with some tendency to increase with distance beyond this (Figure 5 bottom).

Using the successive Chi-square tests on the combined 1988-2006 data, average nest density was statistically lower in arable areas within 0-500m(*P*<0.001), 500-1000m (*P*<0.001) and 1000-1500m (*P*=0.021) than in combined areas further from the nearest trunk A-road (Table 2). When the data were split into 4-5 year periods, average nest density was significantly lower on arable land within 500m of a trunk road than further away in each period, while average density in the 500-1000m distance band was also significantly lower than on land further away from a trunk road in all except the 1993-96 period, but no density differences were detected at any greater distances (Table 2). Equivalent test on individual year nest data were not significnat for any distance bands in early study years, but as the population increased, so statistical power increased and significantly lower nest densities were detected in areas within 500m (2002,2006) or within 1000m (2004,2005) of the nearest trunk road (Table 2).

**Table 2:** Average density (km^-2^) of stone curlew nests on areas (km^2^) of suitable arable land within each band of distance (m) to the nearest Trunk road; together with the upper limit of the maximum distance band for which nest density is statistically lower (Chi-square test *P* value <0.05) than average nest density in the combined higher distance bands (*P* value for each test given in brackets).

		Distance band to nearest Trunk road (km)					
Period	Total nests (N)	<500	500-1000	1000-1500	1500-2000	2000-3000	Max distance (m) with lower nest density
All Years	443	0.061 (<0.001)	0.159 (<0.001)	0.274 (0.021)	0.346 (0.582)	0.370	1500
1988-92	112	0.082 (0.002)	0.108 (0.002)	0.211 (0.063)	0.336 (0.972)	0.338	1000
1993-96	86	0.073 (0.004)	0.164 (0.109)	0.208 (0.200)	0.270 (0.556)	0.320	500
1997-00	88	0.044 (<0.001)	0.134 (0.020)	0.291 (0.892)	0.315 (0.802)	0.294	1000
2002-06	167	0.047 (<0.001)	0.227 (0.004)	0.377 (0.209)	0.444 (0.525)	0.504	1000
2002	22	0.000 (0.030)	0.300 (0.825)	0.333 (0.524)	0.000 (0.012)	0.380	500
2003	24	0.058 (0.085)	0.060 (0.052)	0.277 (0.560)	0.300 (0.541)	0.414	0
2004	29	0.000 (0.013)	0.060 (0.021)	0.388 (0.695)	0.540 (0.549)	0.415	1000
2005	38	0.117 (0.046)	0.120 (0.024)	0.499 (0.809)	0.540 (0.954)	0.533	1000
2006	54	0.058 (0.002)	0.598 (0.728)	0.388 (0.079)	0.839 (0.771)	0.760	500
Area (km^2^) (%)		17.1 (18%)	16.7 (17%0	18.0 (18%)	16.7 (17%)	28.9 (30%))	

### Statistical Modelling in relation to nearby Buildings, Roads and Traffic

The daylight, night and daily total traffic local density variables at any particular value of *s* were almost perfectly correlated with one another. All Spearman rank correlations were >0.99 making it impossible to differentiate between the effects of daylight and night-time traffic on the distribution of stone curlew nests. We therefore restricted later analyses of traffic flow effects to the daily total traffic flow variable, recognising that we could have obtained statistical equivalent and almost identical model relationships if we had used either the daylight or night traffic flow variable instead.

#### Optimum distance-weighted kernel variables

As a first step in model building we fitted model equation 2 to the whole dataset using a combination of one buildings variable and one road/traffic local density variable. The buildings variables assessed were distance to nearest ‘settlement’ and buildings local density based on normal kernels with SD *s* of 250-2000 in steps of 250m. The road/traffic variables assessed were distance to nearest A-road, distance to nearest trunk road and local density of either A-roads, trunk roads or trunk-road traffic, each based on normal kernels with SD *s* of 250-2000 in steps of 250m. The relative fits of the different sets of models, each with optimum *s*, are summarised in Table 3 in terms of the difference (∆AIC) in AIC of any model from the best fitting model (i.e. with minimum AIC).

All models involving the buildings local density variable with *s* of 1000m had much lower AIC (i.e. better fit) than the equivalent model using instead a simpler variable representing distance to the nearest settlement (Table 3). This suggests that the amount of buildings at different distances may have some closer association with nest density than purely the distance away from the nearest ‘settlement’ (which could be anything from a few houses to a town). In all models, the fit was better using the square root of the buildings variable than its untransformed form. The best fitting two-variable model involved the square root of the buildings local density variable (√X_H1000_) with *s* of 1000m and the trunk traffic local density variable (X_TT1000_) with *s* of 1000m. When re-fitted as a quasi-Poisson model, the dispersion parameter *q* estimate was equal to a modest 1.156.

GLM models involving the local density of nearby trunk roads appeared to fit better than those involving the local density of all A-roads. Furthermore, models involving the local density of traffic on the nearby trunk road sections appeared to be slightly better than the equivalent models involving the extent of presence of nearby trunk roads. However, the model involving √X_H1000_ and trunk road presence local density (*s*=1000) was one of the next best forms of model (Table 3). The daily mean traffic flow along sections of trunk roads only varied from 12243 to 21609, a low coefficient of variation relative to the decrease in traffic variable values with the distance of 500m cells from trunk roads. Thus it is difficult with the available information to differentiate with confidence the effects of the presence of nearby trunk roads from the actual level of traffic on them. However, bearing this in mind, we then assessed our best-fit GLM model in further detail.

Amongst all 64 possible models involving the two variables, (square root of) buildings local density and trunk road traffic local density, the model relative likelihoods (given by exp(-Δ_k_AIC/2) [18]) can be used to estimate the model relative likelihoods which suggested that (amongst this subset of models) the best model has relative likelihood of 52%, while alternative models with trunk traffic local density with *s* values of 1250 and 1500m have relative likelihoods of 41% and 4% respectively, suggesting a slightly larger *s* of 1250m for the buildings local density variable would fit almost as well.

**Table 3:** Model AIC increases (∆AIC) from that of the best model (highlighted in italics) of equation 2 form for each of a range of alternative model sets involving one building variable and one road/traffic variable using the optimum normal kernel SD *s* (*s_opt_*).

Buildings variable	*s*_*opt*_	Road/Traffic variable	*s*_*opt*_	∆AIC
Buildings local density	1750	A-road local density	2000	620.9
Buildings local density	1750	Trunk-road local density	1000	421.9
Buildings local density	1750	Trunk traffic local density	1000	388.4
Buildings local density	1750	Distance to trunk road	—	464.5
Buildings local density	1750	Distance to A-road	—	638.7
Square root(Buildings local density)	1500	A-road local density	2000	149.7
Square root(Buildings local density)	1000	Trunk-road local density	1000	17.0
*Square root(Buildings local density)*	*1000*	*Trunk traffic local density*	*1000*	*0.0*
Square root(Buildings local density)	1000	Distance to trunk road	—	44.8
Square root(Buildings local density)	1000	Trunk-road local density	—	181.8
Distance to Settlement	—	Trunk traffic local density	1250	382.2
Distance to Settlement	—	Trunk-road local density	1250	359.6
Square root(Distance to Settlement)	—	Trunk traffic local density	1250	360.9
Square root(Distance to Settlement)	—	Trunk-road local density	1250	337.3
Log(Distance to Settlement)	—	Trunk traffic local density	1000	410.9
Log(Distance to Settlement)	—	Trunk-road local density	1250	385.3
Quadratic (Distance to Settlement)	—	Trunk traffic local density	1250	349.7
Quadratic (Distance to Settlement)	—	Trunk-road local density	1250	324.9
Quadratic (Distance to Settlement)	—	Distance to trunk road	—	581.8

The correlations (r) between the weighted normal kernel variables measuring the local density of nearby buildings and local density of nearby A-roads, trunk roads or trunk roads traffic were all low (< 0.3 for all *s* and *r*(√X_H1000_, X_TT1000_) = 0.08). This indicates that amongst the arable land on suitable soil type within this study region, the amount of nearby buildings is largely unrelated to the amount of trunk roads traffic. Thus in the statistical models it should be possible to distinguish their separate effects on stone curlew nest density.

Table 4 shows the mean observed stone curlew nest density (per km^2^) on arable land over the period 2002-06 in cells classified by their values of X_H1000_ and X_TT1000_ into four or five classes to give roughly equal numbers of observations in each (non-zero-valued) class. Overall average nest density declines with the level of ‘nearby’ buildings and with the level of ‘nearby’ trunk road traffic. In the absence of any nearby trunk road traffic (i.e. X_TT1000_ = 0) and with only the lowest levels of nearby buildings (i.e. X_H1000_ < 7000), average stone curlew nest density over the period 2002-06 was 1.200 per km^2^ (n=284 cells), but as buildings local density increased this declined by 84% to 0.194 per km^2^.

**Table 4:** Average observed stone curlew nest density (per km^2^) in 500m cells classified by buildings local density (X_H1000_) and average daily (March–August) trunk road traffic local density (X_TT1000_) (both with s = 1000m) for the period 2002-06; nest densities weighted by area of suitable arable land per 500m cell (number of cells involved given in brackets).

		Average trunk road daily traffic local density (*X*_TT1000_)				
		0	1-470000	470001-1700000	1700000-5100000	Overall
Buildings local density (*X*_H1000_)	0-7000	1.200 (284)	0.940 (52)	0.865 (58)	0.198 (43)	1.006 (437)
	7001-13000	0.865 (344)	0.182 (37)	0.288 (25)	0.000 (31)	0.716 (437)
	13001-22000	0.542 (333)	0.254 (26)	0.217 (27)	0.083 (20)	0.482 (406)
	22001-44000	0.225 (312)	0.237 (46)	0.178 (40)	0.000 (38)	0.204 (436)
	44001-50000	0.194 (209)	0.318 (40)	0.073 (50)	0.000 (67)	0.157 (426)
	Overall	0.615 (1542)	0.462 (201)	0.379 (200)	0.055 (199)	0.529 (2142)

In areas near only low levels of buildings (i.e. X_H1000_ < 7000), increases in ‘nearby’ trunk road traffic local density are associated with consistent but moderate decreases in nest density. Nest density is consistently very low or zero in the areas of the highest levels of nearby trunk road traffic regardless of the level of nearby buildings (Table 4). For each level of trunk traffic, average nest density was always highest in cells with the least nearby buildings. However, the pattern is not always consistent at intermediate levels of traffic and buildings which may be a result of the geographic spread and clumping of the different combinations of levels of nearby buildings and nearby trunk road traffic.

Further analyses suggested that the best two-variable quasi-Poisson GLM fit involving the square root of buildings local density (√X_H1000_) with s=1000m and trunk road traffic local density (X_TT1000_) with *s*=1000m could be improved by involving the local density of presence of nearby non-trunk A-roads (X_AR250_) with SD=250m (reduction in deviance F test *P* <0.001). Quasi-Poisson models to all years data incorporating the interaction between √X_H1000_ and year (to allow for the possible decline in strength of nest density relationship with buildings local density) did not give significant improvement (reduction in deviance F test *P* =0.78).

#### Allowing for spatial and temporal auto-correlation

However, these initial “model sifting” analyses ignored any effects of spatial auto-correlation and although allowing for inter-year difference in average nest density also ignored any temporal residual auto-correlation.

The effect on model parameters, their standard errors and statistical significance, from potential lack of independence of the nest observation residuals in different years at the same 500m cells was assessed. Specifically, the optimum model was re-fitted using each of a range of assumed inter-year error correlation structures using the Generalised Estimating Equations (GEE) procedure in the SPSS statistics package, treating 500m cells as ‘subjects’ and years as a repeated measures (within-subject) factor. The fits of the assumed model error structures were compared using the quasi-likelihood information criteria (QIC). On assuming a first-order auto-regressive correlation structure between years, the average correlation between model residuals for nest density in successive years at the same 500m cell was only 0.23. Based on minimising QIC, the best fitting model was one assuming independent observations between years within each 500m cell.

With this large dataset, it was not feasible computationally to model temporal and spatial residual auto-correlation simultaneously. However, as one aim was to derive predictions for potential effects of future housing (and other building) developments and increased road traffic, remaining analyses to assess spatial auto-correlation were based on fitting non-temporal GLMM models to data from the most recent period 2002-06 either for individual year’s data or for the period total nest per cell in relation to period-average buildings and road/traffic local density. The additional term (*X*_*AR250*_) for local density of nearby non-trunk A-roads in models of equation 3 form was never significant (all *P* >0.05) when fitted as either a GLMM with exponential decay residual spatial auto-correlation or as a GLM to the most recent period data or individual years (nor was this variable using any other value of *s*). However, in the GLMM involving √X_H1000_ and X_TT1000_ the estimates of the partial effect of both variables was statistically significant (all *P* <0.02) and effectively uncorrelated (all parameters correlations <0.04) whether fitted to total nest numbers over the period 2002-06 or for each year’s nest numbers separately (Table 5). The effect appeared strongest for the buildings local density variable√X_H1000_ (*P* <0.0001 to 0.0034) with the approximate confidence intervals (β ± 1.96SE(β), d.f. = 2138) for its GLMM parameter estimate for any one year encompassing the parameter estimate for any other year. The trunk traffic local density GLMM parameter was slightly less reliably estimated and consistent between individual years (especially for 2006), but was always statistically significant ((*P* <0.001 to 0.020, Table 5). Allowing for spatial auto-correlation reduced the estimates of the size of the effect of each variable and increased the uncertainty (i.e. higher SE and lower *t* and *P*) of the estimates for each year’s data (compare GLMM and GLM fits to 2002-06 total nests data in Table 5). Exponential decay spatial auto-correlation estimates suggest the overall correlation of GLMM model residuals in adjacent cells (i.e. 500m apart) is between 0.10 and 0.28. Model parameters estimates were robust to the choice of spatial auto-correlation function (exponential or Gaussian, Table 5).

**Table 5:** GLMM parameters (α ± SE(α); β ± SE(β), t, P (t)) for model equation type 1 for nest density in relation to the square root of buildings local density (√X_H1000_) and trunk road traffic local density (X_TT1000_ 10^-6^), both with *s*=1000m, for the period 2002-06 fitted to total nests and nests each year separately, using period-average buildings and traffic data; *w* = GLMM parameter estimate for exponential (*r* = *exp*(-*d*/*w*) or Gaussian (*r* = *exp*((-d/*w*)^2^) spatial auto-correlation decay rate.

Period	α ± SE(α)	Buildings √X_H1000_	Trunk traffic X_TT1000_	*w*
		β ± SE(β); t, *P*	β ± SE(β); t, *P*	
2002-06	-2.25 ± 0.24	-0.0082 ± 0.0019; 4.23, <0.0001	-0.863 ± 0.320; 2.70, <0.001	396 m
2002	-4.46 ± 0.31	-0.0072 ± 0.0024; 2.93, 0.0034	-0.863 ± 0.413; 2.09, 0.0369	263 m
2003	-4.10 ± 0.32	-0.0098 ± 0.0027; 3.60, 0.0003	-1.179 ± 0.516; 2.29, 0.0224	222 m
2004	-4.02 ± 0.28	-0.0087 ± 0.0023; 3.81, 0.0001	-1.197 ± 0.464; 2.58, 0.0100	268 m
2005	-3.73 ± 0.27	-0.0107 ± 0.0023; 4.63, <0.0001	-0.900 ± 0.346; 2.60, 0.0094	251 m
2006	-3.63 ± 0.25	-0.0104 ± 0.0021; 4.90, <0.0001	-0.580 ± 0.250; 2.32, 0.0204	240 m
2002-06	-2.35 ± 0.20	-0.0094 ± 0.0017; 5.69, <0.0001	-0.875 ± 0.256; 3.41, 0.0007	Gaussian
				420 m
2002-06	-2.23 ± 0.15	-0.0103 ± 0.0013; 8.20, <0.0001	-0.895 ± 0.188; 4.76, <0.0001	as GLM

## Discussion and Conclusions

The analyses highlight a clear avoidance of buildings and major roads by nesting stone curlew. Nest densities on arable land with the first 500m of settlements (comprising 30% of all arable land) are at least 50% lower than those further away (Table 1). The mechanism by which housing and other buildings or roads directly affects stone curlew population size is unknown, but the effects are so substantial that it is certainly feasible that the avoidance may result in effects on population size, through (for example) increased competition for territories or birds choosing not to breed as suitable habitat is unavailable.

There are therefore consequences for how future development is planned within the region. This research suggests new buildings within 1500m from any arable land suitable for stone curlew to nest on is likely to result in a reduction in nest density in that area. This distance of 1500m has been incorporated within Breckland District [19] and other local Councils’ planning policies to ensure no adverse effect on the stone curlew population. Our models involving √X_H1000_ and X_TT1000_ could be used to try to predict the effect of any proposed spatially-explicit housing development stone curlew nest distribution. Specifically, by adding the proposed new housing numbers onto existing building numbers in the relevant 50m cells, the change in values of buildings local density √X_H1000_ values for each 500m cell can be calculated and then the model used with current and future √X_H1000_ values (and current X_TT1000_ values) to predict the proportional reduction in nest density on suitable arable land for each specific 500m cell. Multiplying these proportions by the observed current nests per 500m cell and summing will give a prediction of the reduction, and especially percentage reduction, in nest numbers arising from the additional housing development [9].

An effect of nearby trunk roads on nest density was detected up to a distance of at least 1000m, and possibly up to 2000m (based on normal kernel SD of 1000m). The 2002-06 data GLM model of Table 5 was used to predict the effect of an anticipated 64% increase in traffic on the A11 trunk road following conversion to dual-carriageway of a section which bisects the Breckland SPA. Using the same type of prediction procedures as proposed above for housing developments, the traffic increase was predicted to increase X_TT1000_ values in nearby 500m cells, lead to reduced nest densities in those cells and lead to a predicted loss of 5.5 nests (3.7%) from current (2002-06 average) levels of 150.4 nests on suitable arable land. Assuming the same model applied to stone curlews on semi-natural grassland habitats the increased traffic would lead to a predicted loss when summed across all 500m cells of 5.1 nests (7.3%) from current 2002-06 average levels of 71 nests on these alternative habitats [20]. The overall predicted loss of 10.6 nests was subsequently used by the Highways Agency, RSPB and Natural England to agree mitigation measures involving bringing of 16ha per predicted lost nest, equal to the creation of 176ha of nesting habitat.

The observed increase over the 19 year period in the spatial stability of the distribution of occupied 500n nest cells could have several contributing causes, such as (i) increased population levels, resulting in increased competition, such that more of the best nest sites tend to be occupied each year, (ii) improvements in farm management or consistency of crop type within arable land may make particular areas consistently most attractive for nesting (iii) quite separately the accuracy of recording nest locations may also have improved over the early years of the study.

One complication in interpreting these results is that the stone curlew data we use for each year is the number of nests and we do not consider breeding success in relation to buildings or roads. We assume that the number of nests is proportional to the number of nesting stone curlews. Individual stone curlews may nest more than once in a given season, particularly if the first attempt fails, for example through predation. The number of nests in a given location may therefore in part be influenced by nest failure rates. This possible confounding factor can be excluded for the effect of proximity to roads on stone curlew nest density because previous work [8] found no effect of proximity to roads on breeding success. From data on the total number of confirmed breeding pairs in each year within the study region, the total nest numbers (analysed here) are on average only 25% higher than the number of confirmed breeding pairs. Hence, the association we observed between nest density and proximity to buildings is too large to be explained even by an extreme negative effect of proximity on failure rates.

Woodlark in Dorset avoided establishing territories in areas with high levels of human disturbance, and therefore the density of territories in such areas was lower (often <50%) than elsewhere [21]. However, density-dependent survival meant woodlark breeding success (fledglings per nesting pair) was higher in those areas where nest density was low, but this effect only partially compensated for the avoidance of suitable nesting habitat due to human disturbance [21]. This example highlights the importance of understanding variations in breeding success in order to fully understand the extent to which population size might be compromised. Both clutch size and fledgling success of great tits declined with the distance and especially level of noise from nearby motorway traffic [22].

Levels of development around sites have been shown to relate to settlement patterns of birds [5] and a range of studies have shown that human population density can be used to predict spatial variation in the threat status of birds [23,24]. The implications of urbanisation, in terms of global biodiversity conservation are considered by McDonald, Karieva and Forman [25]. In general urban areas tend to support lower avian species diversity [26,27]. Bird species that adapt to urban habitats are characterised by traits that include large breeding ranges, high propensity for dispersal, short flight distances when approached by a human, and a life history characterised by high annual fecundity and high adult survival rate [28]. Although stone curlews have a relatively high annual survival rate [2], their other characteristics do not match those of species associated with urban habitats. They have low annual fecundity and large flight distances [7], from which it would be expected that, as observed here, they would avoid built-up areas. However, the magnitude of the avoidance distances involved is surprising.

Our focus has been on arable land because this habitat is likely to be more even in quality across the study area (and also across the period of study) and is likely to be less susceptible to a range of unmeasured factors (such as grazing levels) influencing habitat quality for nesting stone curlews. By focusing on arable land we hope we have in part controlled for variation in habitat quality and we have shown that there is an avoidance of both roads and buildings. We see no reason why birds nesting in semi-natural habitats may not also show a similar avoidance, but it is not necessarily the case that the scale or distance of avoidance is identical.

We have deliberately included all suitable arable land within our analyses, rather than limit ourselves to arable land within the designated boundary of the SPA. Natal and breeding dispersal distances of stone curlews are large [8] and birds hatched or nesting just outside the SPA in one year may well be nesting within the SPA in subsequent years.

We suggest further work exploring the avoidance in relation to buildings and roads would be useful, ideally identifying what the underlying mechanism might be. Avoidance of infrastructure such as roads and buildings is well documented [29], but rarely seems as strong as the effect found here. Based on other studies and species, we suggest potential mechanisms may include increased presence of pets, especially domestic cats, around buildings [30,31]; increased noise around buildings or roads (may be detected [32] or not [33]; light pollution from cars and lorries [8], buildings and/or streetlights; an increased presence of people [34,35]; the presence of predators around infrastructure or at higher densities because of attraction of species such as the red fox to refuse as a source of food [36,37]; or a sensitivity to changes in the habitat/visibility [38,39].

Further work could potentially incorporate building type (for example differentiating residential, industrial/commercial and agricultural) and include this within further analyses. It would also be useful to assess the effect of the intervening habitat type and limitations to visibility between potential nest sites and nearby buildings or major roads. However, in the absence of additional work and an understanding of the mechanisms involved, this study would suggest a clear impact of buildings and major roads, enough to trigger the precautionary principle and suggest that an adverse effect on the SPA is possible from new developments.
